# 26,26,26,27,27,27-Hexadeuterated-1,25-Dihydroxyvitamin D_3_ (1,25D-d_6_) As Adjuvant of Chemotherapy in Breast Cancer Cell Lines

**DOI:** 10.3390/cancers6010067

**Published:** 2013-12-27

**Authors:** Samuel Seoane, Maria A. Bermudez, Juan Sendon-Lago, Anxo Martinez-Ordoñez, Soraya Abdul-Hadi, Miguel Maestro, Antonio Mouriño, Roman Perez-Fernandez

**Affiliations:** 1 Department of Physiology-CIMUS, Endocrine Oncology Laboratories (P1L3), Avda. Barcelona s/n, Campus Vida-University of Santiago de Compostela, Santiago de Compostela 15782, Spain; E-Mails: samuel.seoane@usc.es (S.S.); maria.bermudez@usc.es (M.A.B.); juanjose.sendon@usc.es (J.S.-L.); anxo.martinez.ordonez@rai.usc.es (A.M.-O.); 2 University of Puerto Rico, Recinto de Rio Piedras, Avda. Barbosa-Ponce de Leon, San Juan 23301, Puerto Rico; E-Mail: ahadi92@gmail.com; 3 Department of Organic Chemistry, School of Chemistry, Research Laboratory Ignacio Ribas, Avda. das Ciencias s/n, University of Santiago de Compostela, Santiago de Compostela 15782, Spain; E-Mails: qfmaestr@udc.es (M.M.); antonio.mourino@usc.es (A.M.)

**Keywords:** 1α,25-dihydroxyvitamin D_3_, 26,26,26,27,27,27-hexadeuterated 1α,25-dihydroxyvitamin D_3_, vitamin D, deuterated vitamin D, chemotheraphy, breast cancer

## Abstract

It has been demonstrated that 1,25-dihydroxyvitamin D_3_ (1,25D) and some of its analogues have antitumor activity. 1,25D labeled with deuterium (26,26,26,27,27,27-hexadeuterated 1α,25-dihydroxyvitamin D_3_, or 1,25D-d_6_) is commonly used as internal standard for 1,25D liquid chromatography-mass spectrometry (LC-MS) quantification. In the present study using human breast cancer cell lines, the biological activity of 1,25D-d_6_ administered alone and in combination with two commonly used antineoplastic agents, 5-fluorouracil and etoposide, was evaluated. Using an MTT assay, flow cytometry, and western blots, our data demonstrated that 1,25D-d_6_ has effects similar to the natural hormone on cell proliferation, cell cycle, and apoptosis. Furthermore, the combination of 1,25D-d_6_ and etoposide enhances the antitumoral effects of both compounds. Interestingly, the antitumoral effect is higher in the more aggressive MDA-MB-231 breast cancer cell line. Our data indicate that 1,25D-d_6_ administered alone or in combination with chemotherapy could be a good experimental method for accurately quantifying active 1,25D levels in cultures or in biological fluids, on both *in vitro* breast cancer cell lines and *in vivo* animal experimental models.

## 1. Introduction

In addition to its well known role in calcium homeostasis, numerous studies have demonstrated that 1,25-dihydroxyvitamin D_3_ (1,25D) and its analogues affects other physiological or pathological processes, such as regulation of the proliferation and differentiation of various cell types. This has led to the study of its properties in several processes such as cancer [1,2,3,4]. In breast cancer 1,25D and some of its analogues inhibit cycle progression in MCF-7 human breast adenocarcinoma cells by arresting them in the G0/G1 phase [5,6]. Furthermore, this hormone also induces apoptosis, leading to morphological and biochemical effects such as cell shrinkage, chromatin condensation and DNA fragmentation. In turn, this inhibits tumor cell growth and contributes to tumor suppression [1,7,8]. Most of the above mentioned 1,25D-induced changes in gene expression and other parameters have been characterized by Muñoz *et al.* in several mammary tumor cell lines [9]. They showed that in human breast cancer cells with similar levels of vitamin D receptor (VDR) expression 1,25D treatment induces profound changes in phenotype (*i.e.*, morphology, cytoarchitecture, size), proliferation, sensitivity to apoptotic stimuli, adhesiveness, migration, invasion, and the expression of marker genes associated with the inhibition of myoepithelial characteristics and with decreased malignancy. All of this may contribute to the vitamin’s protective action against neoplasia. 

1,25D and its analogues can also enhance, either synergistically or additively, the antitumor properties of several antineoplastic agents [10]. These properties have been demonstrated specifically in breast cancer cells by combining 1,25D with DNA-damaging agents (*i.e.*, cisplatin and doxorubicin) [11,12], microtubule-disrupting agents (*i.e.*, paclitaxel) [13], topoisomerase inhibitors (*i.e.*, etoposide) [14] and with antimetabolites (*i.e*., 5-fluorouracil) [14]. 

**Figure 1:**
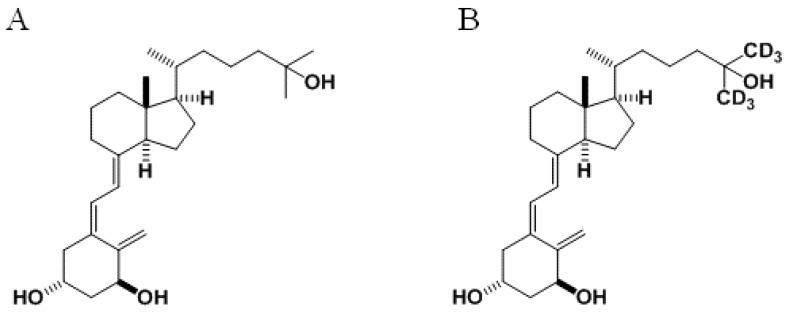
Structures of (**A**) 1,25-dihydroxyvitamin D_3_ (1,25D) and (**B**) its 26,26,26,27,27,27-hexadeuterated analogue (1,25-dihydroxyvitamin D_3_-d_6_; 1,25D-d_6_).

However, the dosage of 1,25D and analogues is a critical factor. Concern over possible side-effects such as hypercalcemia [10,15,16] may have led certain clinical studies to use low dosages that produced disappointing results [17,18,19,20,21,22]. Therefore, the ability to determinate its levels in patients may play an important role in treatment outcome. Deuterated forms of vitamin D are commonly used as internal standards for plasma quantifications by liquid chromatography-mass spectrometry (LC-MS) [23,24]. Thus, administration of 1,25D-d_6_ or a mixture of 1,25D-d_6_ and non-labeled 1,25D could allow accurately quantifying its concentration and bio-availability in an experimental model. One of these deuterated analogs of vitamin D is the 26,26,26,27,27,27-hexadeuterated 1,25-dihydroxyvitamin D_3_ (1,25D-d_6_) (Figure 1A,B). 

To compare the effects of 1,25D and its deuterated form (1,25D-d_6_) on human breast cancer cells, these compounds were administered alone and in combination with two antineoplastic agents, 5-fluorouracil and etoposide, and then citotoxicity, cell cycle, apoptosis, and three-dimensional cell growth was evaluated.

## 2. Results and Discussion

**Figure 2:**
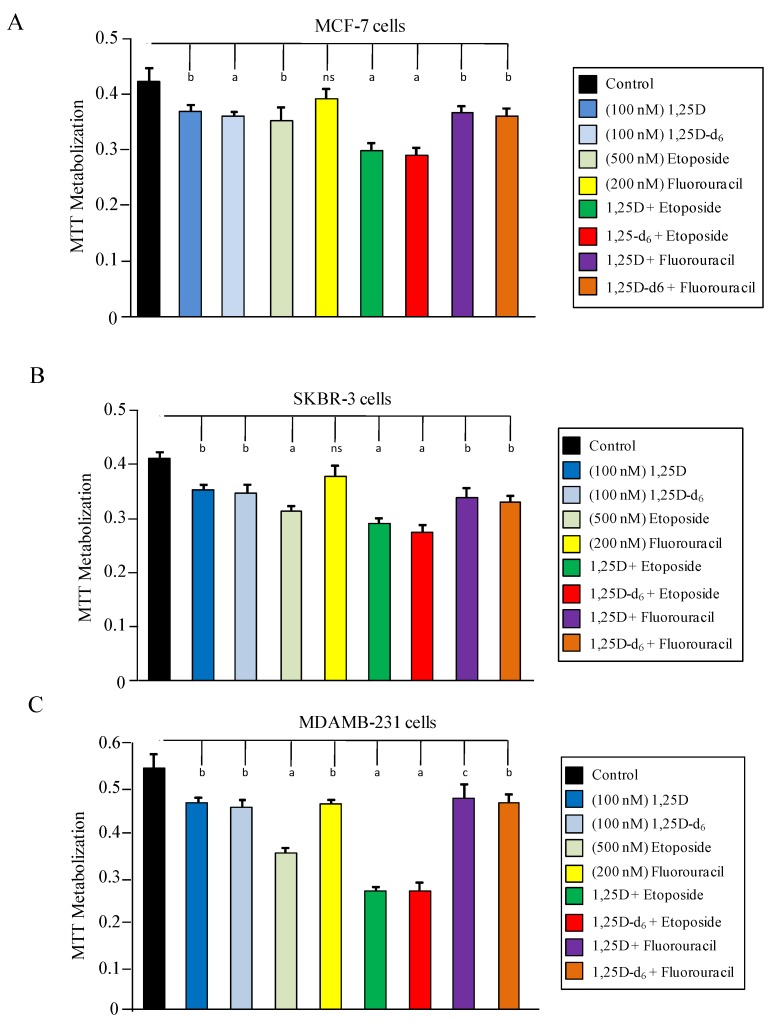
Cell viability in (**A**) MCF-7 cells; (**B**) SKBR-3 cells, and (**C**) MDA-MB-231 cells 48 h after administration of placebo (control), 100 nM 1,25D, 100 Nm 1,25D-d_6_, 500 nM etoposide, 200 nM 5-fluorouracil, and the combination of these substances at the same doses. The values represent means ± SD from three independent determinations. a = *p* < 0.001 *vs.* control cells; b = *p* < 0.01 *vs*. control cells; c = *p* < 0.05 *vs.* control cells; ns = not significant.

To evaluate cell viability after 1,25D, 1,25D-d_6_, 5-fluorouracil, and etoposide administration, the human breast cancer lines were treated as described in the Experimental section, followed by an MTT assay at 48 h. We found that 1,25D-d_6_ produced effects similar to the natural hormone (1,25D), inducing significantly decreased cell viability in all cell lines, in relation to control cells (*p* = 0.001 in MCF-7 cells, *p* = 0.0007 in SKBR-3 cells, and *p* = 0.002 in MDA-MB-231 cells; Figure 2A–C). Combination of 1,25D-d_6_ with etoposide enhanced the effect on cell viability of each compound in all cell lines (1,25D-d_6_ + etoposide *vs.* 1,25D-d_6_ or etoposide, MCF-7 cells: *p* = 0.0002 and *p* = 0.002, respectively; SKBR-3 cells: *p* = 0.003 and *p* = 0.02, respectively; and MDA-MB-231 cells: *p* = 0.000006 and *p* = 0.003, respectively). 

As shown in Figure 2C, decreased cell viability is greater in the more aggressive breast cancer MDA-MB-231 cell line than in MCF-7 or SKBR-3 cells. Administration of 5-fluorouracil plus either 1,25D or 1,25D-d_6_ does not significantly reduce cell viability as compared with administration of each compound alone (Figure 2A–C). Decreased cell viability in the more aggressive cell line MDA-MB-231 after treatment with etoposide plus 1,25D or 1,25D-d_6_ could not be explained by vitamin D receptor (VDR) levels because these cells had lower VDR expression, compared with the other less aggressive MCF-7 and SKBR-3cell lines [25]. Lower VDR expression has also been demonstrated in invasive tumors as compared with normal tissue [26]. VDR levels are very important to inducing 1,25D-dependent anti-proliferative effects on breast cancer cells, but overexpression of VDR in MDA-MB-231 cells does not restores the sensitivity to the 1α-hydroxyvitamin D_5_ analogue, as occurs in cells with high levels of VDR, suggesting that other factors are involved in 1,25D-mediated inhibition of cell proliferation [27]. 

**Figure 3:**
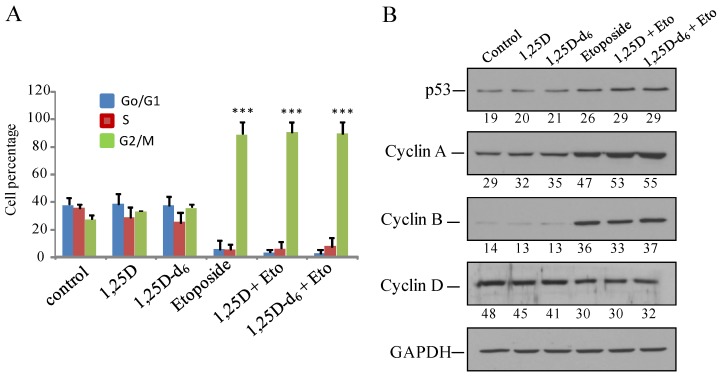
(**A**) Flow cytometry analysis of cell cycle in MDA-MB-231 cells after 48 h of treatment with placebo (control), 100 nM 1,25D, 100 nM 1,25D-d_6_, 500 nM etoposide, and 1,25D or 100 nM 1,25D-d_6_ (100 nM) + etoposide (Eto, 500 nM). The values represent means ± SD from three independent determinations.*** = *p* < 0.001; (**B**) Western blot of p53, cyclin A, cyclin B, cyclin D and GAPDH (used as loading control) from protein extracts obtained from MDA-MB-231 cells treated as in (**A**). Numbers show the quantification of protein expression after normalization to GAPDH. A representative experiment is shown.

Given that our results show that MDA-MB-231 cells are the most responsive to treatment with 1,25D-d_6_ plus etoposide, we focused our next experiments on this cell line and this antineoplastic agent. The lower cell viability in MDA-MB-231 cells after treatment with 1,25D and 1,25D-d_6_ plus etoposide could be explained by reduced cell proliferation or increased cell death. Therefore, we next investigated the effect of both hormones and etoposide on cell cycle and apoptosis. It is well known that etoposide is cell-cycle dependent and phase specific, inducing G2/M arrest and activation of p53 [28,29]. Our results corroborate this data. Figure 3A shows that administration of etoposide increased the G2/M phase in relation to control cells (*p* < 0.001). In addition, Western blot indicated that etoposide also increased cyclin A, cyclin B, and p53 expression (Figure 3B), suggesting that decreased cell proliferation is due, at least in part, to alterations in cell cycle. Administration of etoposide plus 1,25D or 1,25D-d_6_ modifies neither G2/M phase of the cell cycle or p53, cyclin A, and cyclin B expression as compared with the administration of etoposide alone (Figure 3A,B).

**Figure 4:**
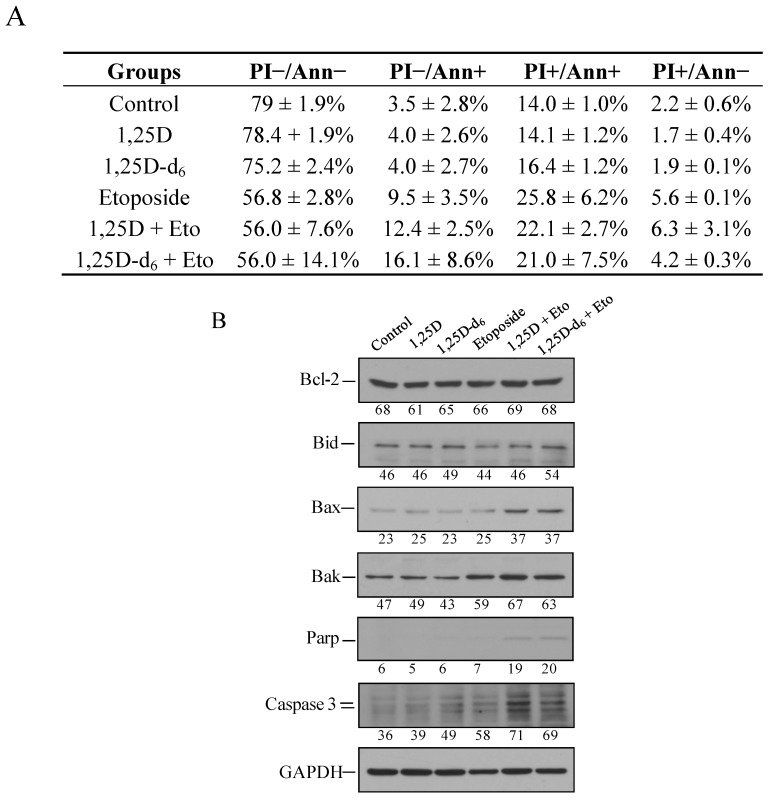
(**A**) Flow cytometry analysis using annexin-FICT and Propidium Iodide (PI) indicated increased apoptosis (PI−/Ann+ and PI+/Ann+) of MDA-MB-231 cells 48 h after administration of etoposide, and etoposide plus 1,25D, or 1,25D-d_6_ as compared to control cells. The values represent means ± SD from three independent determinations; (**B**) Western blot analyses of pro- (Bid, Bax, Bak, cleaved PARP, and active caspase-3) and anti- (Bcl-2) apoptotic proteins in MDA-MB-231 cells treated as in Figure 3A. Numbers show the quantification of protein expression after normalization to GAPDH. A representative experiment is shown.

Our next step was to evaluate cell death by flow cytometry. As shown in Figure 4A, administration of etoposide alone to the MDA-MB-231 cells induced an increased early (PI−/Ann+: 9.5 ± 3.5%) and late (PI+/Ann+: 25.8 ± 6.2%) apoptosis with respect to control cells (3.5 ± 2.8%, not significant, and 14.0 ± 1.0%, *p* = 0.03, respectively). Administration of 1,25D plus etoposide (PI−/Ann+: 12.4 ± 2.5%, PI+/Ann+: 22.1 ± 2.7%) significantly enhances the effect on apoptosis with respect to administration of 1,25D (PI−/Ann+: 4 ± 2.6%, *p* = 0.02, PI+/Ann+: 14.1 ± 1.2%, *p* = 0.009, respectively), but not with respect to administration of etoposide alone. Administration of 1,25D-d_6_ plus etoposide enhances, but not significantly, early apoptosis with respect the administration of each compound (1,25D-d_6_ + etoposide versus 1,25D-d_6_ or etoposide, *p* = 0.081 and *p* = 0.3, respectively). We also used Western blot to analyse the expression levels of several proteins involved in the apoptosis pathway. Our data indicates that no significant changes were found in the anti-apoptotic Bcl-2 and the pro-apoptotic Bid proteins after administration either alone or in combination of etoposide, 1,25D, and 1,25D-d_6_, in relation to control cells (Figure 4B). Administration of etoposide alone or combined either with 1,25D, or 1,25D-d_6_ increased the pro-apoptotic Bak protein. In addition, a visible increase in Bax, cleaved PARP, and active caspase-3 proteins after etoposide plus 1,25D, or 1,25D-d_6_ was observed, as compared with control cells and cells treated with 1,25D, 1,25D-d_6_, or etoposide alone (Figure 4B). Thus, our data seems to indicate a increased cell death when the MDA-MB-231 cells are treated with etoposide plus 1,25D or 1,25D-d_6_. This cell death was clearly related to an increased early apoptosis, as shown by PI−/annexin V+ cells, and induction of pro-apoptotic markers, such as active caspase-3, cleaved PARP, Bax and Bak. Several reports have described the anti-tumor effects of 1,25D on cancer cells by regulating key mediators of apoptosis [10], suggesting that the most probable mechanisms are through the downregulation of the anti-apoptotic protein Bcl-2 [30], and disruption of mitochondrial function, which is associated with Bax translocation to mitochondria, cytochrome *c* release, and production of reactive oxygen species [31]. In addition, 1,25D-induced apoptosis via caspase-independent mechanisms has also been described [32]. Our results support the above mentioned caspase-dependent mechanism when either 1,25D or 1,25D-d_6_ are combined with etoposide.

**Figure 5:**
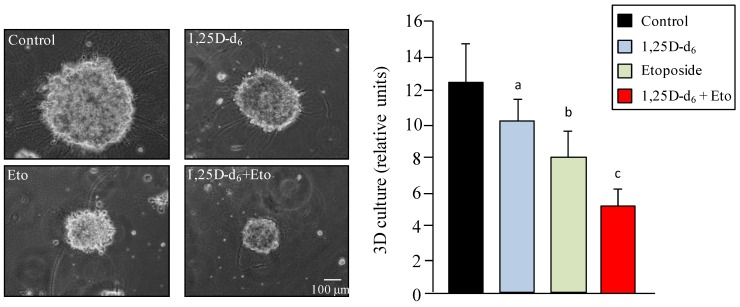
Three-dimensional growth of MDA-MB-231 cells 48 h after treatment with placebo (control), 100 nM 1,25D-d_6_, 500 nM etoposide, and 100 nM 1,25D-d_6_ plus 500 nM etoposide. The values represent means ± SD from three independent determinations. a = *p*< 0.05 *vs.* control; b = *p* < 0.001 *vs.* control; c = *p* < 0.01 *vs.* etoposide.

We also explored three-dimensional growth of MDA-MB-231 cells. Figure 5 shows that treatment with etoposide produced a significant decrease (*p* = 0.0007) in sphere diameter compared with control cells, and this effect increased when etoposide was combined with 1,25D-d_6_ (*p* = 0.002). 

Previous studies have reported that some deuterated vitamin D analogs have similar or lower effects than their non-deuterated forms on clonal growth and cell differentiation in several leukemia cell lines, in addition to stimulating intestinal calcium absorption and bone calcium mobilization [33]. However, other studies have demonstrated deuterated 1,25D to be more potent at lower concentrations than 1,25D on growth plate cartilage [34]. A recent study demonstrated that, when deuterated, the gemini vitamin D analogues (derivatives of 1,25D with two chains emanating at C20) showed higher bioactivity, probably by stabilizing the ligand binding domain (LBD) of the VDR and by enhanced coactivator interactions [35]. Our data do not reveal significant differences in bioactivity between 1,25D and 1,25D-d_6_ when combined with etoposide. It has been demonstrated that etoposide inhibits the ability of 1,25D to cause accumulation of 25-hydroxyvitamin D_3_ 24-hydroxylase mRNA(CYP24), an enzyme that catabolizes this hormone, thus increasing the bioavailability of 1,25D [36]. Although we did not evaluate CYP24 mRNA levels, we found that etoposide in combination with 1,25D-d_6_ had similar effects to 1,25D plus etoposide on cell viability, cell proliferation, and apoptosis. Thus, it seems reasonable to assume that similar mechanisms are involved with 1,25D-d_6_, and to speculate that etoposide could also increase its bioavailability and providing it with similar biological activity, at least in the mammary tumor cell lines used in our experimental approach.

In summary, our results seems to indicate that administration of etoposide (and perhaps other antineoplastic agents) plus 1,25D-d_6_, instead of the non-deuterated form, could be a better method for accurately quantifying its concentration in breast cancer cell lines or in animal experimental models *in vivo*. 

## 3. Experimental Section

### 3.1. Cell Culture and Drugs

The human breast carcinoma cell lines MCF-7, SK-BR-3, and MDA-MB-231, obtained from the European Collection of Cell Cultures (Salisbury, Wilts, UK), were grown in 90 mm petri dishes in DMEM supplemented with 10% fetal bovine serum (FBS), 100 U/mL penicillin, 100 mg/mL streptomycin, and 2 mM L-glutamine in an air-CO_2_ (95:5) atmosphere at 37 °C. Confluent cells were washed twice with PBS and harvested by a brief incubation with trypsin-EDTA solution (Sigma-Aldrich, Madrid, Spain) in PBS. 1,25D and 1,25 D-d_6_ were provided by Profs. Antonio Mouriño and Miguel Maestro (University of Santiago de Compostela, Santiago de Compostela, Spain). 5-Fluorouracil and etoposide were obtained from Ferrer Farma (Barcelona, Spain) and Teva Genéricos Española, S.L. (Madrid, Spain). 5-Fluorouracil and etoposide were dissolved in PBS and used at 200 nM and 500 nM, respectively. 

### 3.2. Treatments

Cell lines were treated either with 1,25D or 1,25D-d_6_ at 100 nM. The two antineoplastic agents, etoposide and 5-fluorouracil were used at 500 nM and 200 nM, respectively. The same doses were used when combined with 1,25D or 1,25D-d_6_.

### 3.3. MTT Metabolization

Cell viability experiments were carried out using MTT assay. Cells (2.5 × 10^4^ cells/mL) were seeded in a volume of 0.5 mL into 24-well tissue culture plates. MCF-7, SKBR-3, and MDA-MB231 cell lines were treated after 24 h as described above. The absorbance of the samples was recorded 48 h after treatment at 570 nm in a multiwell plate reader (LB 940 Mithras, Berthold Technologies, Bad Wildbad, Germany). Results were plotted for each cell line as the mean ± SD values of quadruplicates from at least two independent experiments.

### 3.4. Cell Cycle and Apoptosis Assays

Cell cycle and apoptosis assays were carried out by using a Guava flow cytometer (Millipore Corporation, Billerica, MA, USA). Briefly, 2 × 10^5^ cells/well were cultured in DMEN. Forty-eight hours later, cell were harvested, fixed with 70% cold ethanol for 30 min, washed with PBS, and incubated with ribonuclease (100 µg/mL), and propidium iodide (PI, 50 µg/mL) for 60 min in darkness, for cell cycle evaluation. Apoptosis analyses were performed using Annexin V (Ann)-FITC. Cells (2 × 10^5^) were harvested, washed twice with PBS, and resuspended in 1 × binding buffer (10 mM Hepes (pH 7.4), 140 mM NaCl, and 2.5 mM CaCl_2_). 5 µL of FITC-Annexin V was added and incubated for 15 min at room temperature in darkness. Finally, 400 µL of 1 × binding buffer was added to each tube, and analyzed. 

### 3.5. Western Blot Assays

Western blotting of MDA-MB231 cells was performed as previously described [37]. Briefly, 50 µg of total protein was subjected to SDS–PAGE electrophoresis. Proteins were transferred to a nitrocellulose membrane, blocked, and immunolabeled overnight at 4 °C with a primary antibody, and then incubated with the appropriate secondary antibody. The signal was detected with the Pierce ECL Western blotting substrate (Thermo Scientific, Rockford, IL, USA) and visualized by putting the blot in contact with standard X-ray film following the manufacturer’s instructions. The following antibodies were used: anti-cleaved PARP, anti-Bcl-2, anti-Cyclin D, anti-p-53, and anti-GAPDH (Santa Cruz Biotechnology, CA, USA), anti-active Caspase-3, anti- Cyclin A, and anti-Cyclin B (BD Biosciences, San Diego, CA, USA), and anti-Bid, anti-Bax and anti-Bak (Cell Signaling Technology, Danvers, MA, USA).

### 3.6. Three-Dimensional Cell Culture

For 3D cell culture, 12 mm coverslips were coated with 60 µL of ice-cold Matrigel (BD Biosciences) and incubated at 37 °C for 20 min to allow the Matrigel to solidify. Cells were treated for 5 min with 0.25% trypsin-EDTA solution (2.5 g/L of trypsin, 0.38 g/L of EDTA; Sigma, Madrid, Spain). A single cell suspension containing 5 × 10^3^ cells per 100 µL of culture medium, supplemented with 2% (vol/vol) of Matrigel, was carefully placed on the coverslips on top of the solidified Matrigel and incubated at 37 °C for 30 min. Coverslips were then placed in six-well plates with 500 µL of culture medium per well. After 10 days, cells were treated with 1,25D-d_6_ and etoposide alone or in combination for one week. Photographs of the 3D cultures were taken with a Nikon Eclipse Ti-S inverted microscope (Izasa, Barcelona, Spain) equipped with a ProgRes C3 camera and the ProgRess Capture Pro 2.7 software [38]. Quantification of the sphere diameters was made manually by tracing a straight line across the diameter of the sphere and scoring its value as arbitrary length units. 50 spheres were scored for each condition to calculate the mean. 

### 3.7. Statistical Analysis

Each experiment was performed at least three times. Values are expressed as mean ± standard deviation. Means were compared using one-way ANOVA, with the Tukey’s test for post hoc comparisons. *p* values of less than 0.05 were considered statistically significant. The MATLAB R2011a Version7.1 software [39] was used for all calculations. 

## 4. Conclusions

Our data indicates that 1,25D-d_6_ had similar bioactivity to the natural hormone 1,25D when combined with the antineoplastic drug, etoposide. Combined with 1,25D-d_6_, etoposide enhanced the antitumoral activity of each drug on cell viability in the breast cancer cell lines MCF-7, SKBR-3 and MDA-MB-231. Interestingly, the antitumor effect was higher in the more aggressive cell line MDA-MB-231. Our results support that 1,25D-d_6_ administered alone or in combination with chemotherapy could be a good experimental method for accurately quantifying its concentration in culture medium or biological fluids. 
